# Patients with skin smear positive leprosy in Bangladesh are the main risk factor for leprosy development: 21-year follow-up in the household contact study (COCOA)

**DOI:** 10.1371/journal.pntd.0008687

**Published:** 2020-10-30

**Authors:** Emily E. V. Quilter, C. Ruth Butlin, Surendra Singh, Khorshed Alam, Diana N. J. Lockwood

**Affiliations:** 1 Department of Infectious and Tropical Diseases, London School of Hygiene and Tropical Medicine, Keppel street, London, United Kingdom; 2 The Leprosy Mission England and Wales, Peterborough, United Kingdom; 3 The Leprosy Mission International, Nilphamari, Bangladesh; Johns Hopkins University, UNITED STATES

## Abstract

**Background:**

Leprosy transmission is ongoing; globally and within Bangladesh. Household contacts of leprosy cases are at increased risk of leprosy development. Identification of household contacts at highest risk would optimize this process.

**Methods:**

The temporal pattern of new case presentation amongst household contacts was documented in the COCOA (Contact Cohort Analysis) study. The COCOA study actively examined household contacts of confirmed leprosy index cases identified in 1995, and 2000–2014, to provide evidence for timings of contact examination policies. Data was available on 9527 index cases and 38303 household contacts. 666 household contacts were diagnosed with leprosy throughout the follow-up (maximum follow-up of 21 years). Risk factors for leprosy development within the data analysed, were identified using Cox proportional hazard regression.

**Findings:**

The dominant risk factor for household contacts developing leprosy was having a highly skin smear positive index case in the household. As the grading of initial slit skin smear of the index case increased from negative to high positive (4–6), the hazard of their associated household contacts developing leprosy increases by 3.14 times (p<0.001). Being a blood relative was not a risk factor, no gender differences in susceptibility were found.

**Interpretation:**

We found a dominance of a single variable predicting risk for leprosy transmission–skin smear positive index cases. A small number of cases are maintaining transmission in the household setting. Focus should be performing contact examinations on these households and detecting new skin smear positive index cases. Conducting slit-skin smears on new cases is needed for predicting risk; such services need supporting. If skin smear positive cases are sustaining leprosy infection within the household setting, the administration of single-dose rifampicin (SDR) to household contacts as the sole intervention in Bangladesh will not be effective.

## Author summary

The number of newly detected leprosy cases worldwide has remained stable over the last ten years. Household contacts of leprosy cases are at increased risk of leprosy development. Household contact examinations and case detection are critical aspects of control. Specific risk factors (recognizable by field staff) for leprosy development need to be identified to aid these control efforts. Targeted household examinations could increase early case detection: this is important because untreated leprosy cases are sustaining transmission.

This is the largest, most comprehensive study of development of leprosy in household contacts conducted recently with a maximum follow-up of 21 years. We found a single dominant risk factor for the development of leprosy in the household setting, namely slit skin smear positivity in the index case. This is important for leprosy control because household contact examinations can be focused on contacts at highest risk. Implementation would be simple as the risk factor is identified by leprosy staff in the field. Prioritising households known to be at highest risk of leprosy development would allow efficient allocation of resources. Our results cast doubt on the success of administering the recommended prophylactic intervention for contacts; the use of this intervention needs to be re-evaluated.

## Introduction

Leprosy is a chronic, granulomatous disease caused by the intracellular bacterium *Mycobacterium leprae (M*. *leprae)*.[1, 2] An infection of the skin and peripheral nerves, [3, 4] the incubation period is variable—from months up to 30 years. [2] The main route of infection is via the airways/ oronasal mucous membranes. [2] Leprosy is microbiologically curable; early case detection and treatment with multi-drug therapy (MDT) reduces risk of risk of onward transmission and development of disability. [5] Cure rates with modern MDT are very high and relapse rates are low. [2, 6] Leprosy has significant social and psychological implications. [7] Stigma is an important cause of delayed diagnosis. [8, 9] Leprosy exhibits a clustered distribution at the household (HH)/ social interaction level. [10]

Around 210,000 new cases are detected annually globally, with little change over the last ten years. [11] Currently 80% of newly detected cases occur within five countries. [1, 3] Bangladesh ranks fifth globally amongst newly detected cases. [12, 13]

The epidemiology of leprosy and mode of transmission of infection remains poorly understood. The main route is respiratory transmission with nasal secretions containing *M*. *leprae* from untreated patients with leprosy. [1, 14] Patients with a positive slit-skin smear [1] are thought to be the most infectious—this includes multibacillary (MB) classification and LL, BL and some BB cases as classified on Ridley Jopling spectrum. [2] In countries endemic for leprosy, up to 5% of the population are chronic carriers of *M*. *leprae* DNA in their noses without developing clinical disease. [4]

Several risk factors for the development of leprosy in HH contacts have been identified, however their independent contributions are not yet quantified. [15, 16] Close and prolonged contact with a leprosy case, particularly at HH level is a risk factor for infection. [15] Untreated MB and lepromatous leprosy patients are thought to be the most important source of transmission. [17] Physical distance (living in the same HH) and genetic distance (first degree relative), duration of exposure to index case [17] and age of HH contact are independently associated with risk of leprosy development. [16] Blood relations to index cases are at a higher risk of acquiring leprosy compared to non-blood relations dwelling within the same HH [17] which could be due to genetic susceptibility. [2] HH size has been reported as a risk factor amongst HH contacts living in HHs greater than seven people. [18] Possible explanations include overcrowding and correlation with lower Socio-Economic Status (SES) amongst larger HHs. [5, 18] Low family income, reduced access to clean water and other unfavourable socio-economic indicators have been associated with the development of leprosy compared to controls. Environmental and zoonotic reservoirs have been described as modes of transmission of infection, however their significance in terms of case numbers is not fully understood. [19]

Leprosy programmes utilize contact examinations targeting HHs/ social clusters for case detection which is expensive and can be inefficient. [20] Early case detection and treatment reduces further transmission of infection and helps prevent development of Grade-2 disabilities. [5] The latest WHO global leprosy strategy focuses on active case finding activities in high-endemic areas through systematic tracing and examination of HH contacts and neighbours. [21] Introducing a targeted approach to household contact examination and administration of prophylaxis, which prioritizes HH contacts known to be at highest risk developing leprosy, would optimize case detection. Specific risk factors (recognizable by field staff) therefore need to be identified.

Follow-up (FU) of HHs with leprosy cases has been routinely carried out in a leprosy control unit in North-West (NW) Bangladesh since before 1995. The Leprosy Field Research in Bangladesh (LFRB) project is a leprosy control programme covering four districts in NW-Bangladesh with a total population of approximately 7.5 million; the project works as part of the non-governmental organisation The Leprosy Mission International, Bangladesh (TLMIB). Data from this routine surveillance period was used with further data from a cross sectional survey completed in 2015/16. Compared to the rest of the country, NW-Bangladesh has a high PB rate, and has similar climate, environment and geography. This high PB rate is probably due to the intervention effect; the area has a well-established leprosy control unit engaged with leprosy research for over 20 years.

The COCOA (Contact Cohort Analysis) study actively examined all HH contacts (anyone permanently living at same house, eating together, at least past six-months) of confirmed leprosy index cases identified in the four districts in 1995, and 2000–2014, to provide evidence for timings of contact examination policies. [22, 23] Prevalence rates fell markedly between the 1995 cohort and later years. The registered prevalence rate per 10 000 population was 4.57 in 1995 and 0.78 in 2015. [24] Despite the absolute number of new cases in annual cohorts changing, the spectrum of cases was similar. [23] Index cases were defined as the first person from the HH registered in the leprosy control unit as a new case during relevant period (1995, or 2000–2014) who was enrolled in the study.

The data from the original COCOA study was used to test hypotheses that the risk of HH contacts of confirmed leprosy cases developing leprosy is associated with characteristics of the index case, in particular leprosy type, level of initial skin smear and sex. We investigated whether risk is associated with specific HH contact features such as blood relative, age and sex. Through this analysis we aimed to identify a specific, dominant risk factor for leprosy development which can be used to prioritise and focus regular HH contact examinations and the administration of prophylaxis to contacts at highest risk.

## Methods

The COCOA study was designed as a two-part study as described by Butlin et al. [23] Firstly, a retrospective analysis of data from 1995 and 2000–2014 of annual HH contact examinations during routine surveillance period (two years after paucibacillary (PB) index case diagnosis and up to five years after MB index case diagnosis) was conducted within the study area. Data was extracted from clinic records. Furthermore, in 2015–16 (study period) a cross-sectional study of HH contacts of index cases diagnosed between the aforementioned years was conducted. This was an additional one-off examination to provide additional data from later follow-up years. Our data includes patients who presented voluntarily in between house visits so they were allocated to year of registration.

Suspect cases (anyone with suggestive signs of leprosy) were initially identified by staff with minimum six-months training as leprosy control assistants, and treated immediately, once a doctor confirmed the diagnosis and classification. Suspect cases received a full body visual examination, sensory testing of patches, palpation of peripheral nerves at sites of predilection and nerve function assessment for impairment. [2, 5] A slit-skin smear was performed on those who consented. [5] If diagnosis remained uncertain, the contact was observed for three-six months. If uncertainty remained, then skin biopsies were performed and a second opinion from a dermatologist/ neurologist sought. All index cases who had HH contacts and were diagnosed within the defined time period and geographical location were considered for inclusion. On repeat visits to the HH for HHC examination, staff did clinical assessments of old index cases to exclude relapse or new disability requiring intervention, however no repeat slit-skin smear tests were conducted on index cases as none showed suspicion of relapse.

Any new case registered in the specified time period, or HH contact, not willing to participate were excluded from the study. HH contacts of confirmed index cases who had previously received single-dose rifampicin (SDR) in the COLEP trial [20] or contacts of index cases who had participated in the non-standard MDT regimen (6m MBMDT) trial were also not included because it was expected that these interventions would affect incidence rates.

### Data handling and analysis

The data was collected on paper forms, entered into a custom Access database then exported into Excel spreadsheets and saved in STATA format for analysis using STATA 16. Data was cleaned and consistency checks performed between related variables with appropriate corrections applied.

To identify significant risk/ protective factors cox proportional hazard regression analysis was conducted. This is a statistical method of investigating the effect of one or more variables upon the time a specific outcome occurs. Variables found to be significant in univariate analysis were included in additional multivariate analysis and stepwise analysis procedure. Reported here are variables with statistically significant hazard ratios identified as risk/ protective factors for leprosy diagnosis amongst HH contacts of confirmed index cases. Applying stepwise procedure identifies the optimal subset of predictive variables. The complete dataset is publicly available from the BioStudies Submission Tool database.

No additional ethical approval was required for the completion of this secondary data analysis. Ethical approval for the original COCOA study was approved by the Institutional Review Board, The Leprosy Mission International, Bangladesh on 13^th^ May 2014 and was sufficient for the completion of this study, conducted as part of a MSc project at LSHTM.

## Results

A total of 18211 new leprosy cases were registered in 1995 and 2000–2014 within the leprosy control programme in NW Bangladesh. Exclusion criteria was applied where the index case was involved in other research projects, potentially affecting the transmission of leprosy. Other reasons for exclusion included the new case was already listed as a contact of an earlier case already enrolled, or that no-one from the original HH was still resident in that address. An additional 5713 index cases were not assessed due to time constraints. Data on 9527 index cases and their associated HH contacts was available for analysis. Table 1 shows the variables recorded for the index cases assessed and included in study.

**Table 1:** Frequencies and percent of recorded variables amongst index cases.

Variable		Frequency	Percent (%)
**Sex**	**Male**	5514	57.88
	**Female**	4013	42.12
	**Total**	**9527**	**100**
**Age group at diagnosis**	**0–15**	1545	16.22
	**16+**	7982	83.78
	**Total**	**9527**	**100**
**Leprosy group**	**PB (male)**	7608 (4158)	79.86 (54.64)
	**MB**^a^ **(male)**	1919 (1356)	20.14 (70.66)
	**Total**	**9527**	**100**
**R-J classification**^b^	**I/TT/BT/PN**	8901	93.43
	**BB/BL/LL**	626	6.57
	**Total**	**9527**	**100**
**WHO grade**^c^	**0**	8406	88.25
	**1**	569	5.97
	**2**	550	5.77
	**Total**	**9525**	**100**
**Initial smear of index case**^d^	**0**	8068	93.44
	**1–3 (low positive)**	179	2.07
	**4–6 (high positive)**	387	4.48
	**Total**	**8634**	**100**
**Index duration of symptoms (months)**	**0–12**	6806	71.48
	**13–24**	1319	13.85
	**25+**	1396	14.66
	**Total**	**9521**	**100**

^a^MB classification—more than five skin lesions/ more than one peripheral nerve affected/ smear positive. All other leprosy cases defined as PB.

^b^R-J classification (Ridley-Jopling)–an alternative classification system based on the clinical symptoms and immunological response to disease classifying patients into five member groups: Tuberculoid (TT), Borderline Tuberculoid (BT), Borderline (BB), Borderline Lepromatous (BL) and Lepromatous. PB leprosy includes TT and some BT, MB leprosy includes LL, BL BB cases, and some BT cases. [25] The RJ classification has now been modified and includes an Indeterminate category [26], as used in this study.

^c^A disability grading system used both as an indicator for early case detection and physical impairment of leprosy patient.

^d^Slit-skin smear is a diagnostic technique performed on leprosy patients detecting *M*. *leprae* acid fast bacilli reported as Bacteriological Index (BI) on a logarithmic scale of zero to six. For the purposes of this analysis, Index smear was categorized into three groups; smear negative, low positive (1–3) and high positive (4–6).

^e^Data on index duration of symptoms (before diagnosis, i.e. detection delay) was self-reported.

There was a male predominance of index cases (57.88%) and a huge excess of PB cases (79.86%) which is typical of the leprosy picture in Bangladesh. [11] Considering the Ridley- Jopling classification [26] 93.43% had indeterminate (I), tuberculoid (TT), borderline tuberculoid (BT) or pure neuritic (PN), this is a high rate of these Ridley-Jopling types and a low rate of the borderline-borderline (BB), borderline lepromatous (BL) and lepromatous (LL) types. 16.22% of index cases were under the age of 15 years at diagnosis indicating ongoing leprosy transmission. A low rate of WHO disability was observed (Table 1) with only 5.77% having WHO grade 2 disabilities. 4.48% of index cases had a highly positive skin smear and 93.44% had negative skin smears. 71.48% of index cases had symptoms for less than 12 months. (Table 1) Among adult index cases 79.2% were PB, and among child index cases– 89.7% were PB as described by Butlin et al. [22]

Data on 38303 HH contacts (50.73% male, 54.81% aged 16+) of known leprosy index cases, who were initially leprosy free at the start of the study, were analysed. A total of 666 HH contacts were diagnosed with leprosy throughout the FU– 50.45% female, 62.16% adult. Tables 2 and 3 show the frequency of variables and statistically significant findings based on chi-squared tests of association.

**Table 2:** Results of FU for HH contacts for all related variables, including Chi^2^ test results.

Variable		Number of HH contacts		Total (%)	Chi^2^	P value
		No leprosy (%)	Leprosy (%)			
**Sex of Index case**	**Male**	22189 (98.06)	438 (1.94)	22627 (100)	12.56	<0.001
	**Female**	15448 (98.55)	228 (1.45)	15676 (100)		
	**Total (%)**	**37637 (98.26)**	**666 (1.74)**	**38303 (100)**		
**Sex of HH contact**	**Male**	19102 (98.3)	330 (1.7)	19432 (100)	0.379	0.538
	**Female**	18535 (98.22)	336 (1.78)	18871 (100)		
	**Total (%)**	**37637 (98.26)**	**666 (1.74)**	**38303 (100)**		
**Age of HH contact at Index case diagnosis (years)**	**0–15**	17057 (98.54)	252 (1.46)	17309 (100)	14.79	<0.001
	**16+**	20580 (98.03)	414 (1.97)	20994 (100)		
	**Total (%)**	**37637 (98.26)**	**666 (1.74)**	**38303 (100)**		
**Leprosy group of index case**	**PB**	30376 (98.92)	332 (1.08)	30708 (100)	391.99	<0.001
	**MB**	7261 (95.6)	334 (4.4)	7595 (100)		
	**Total (%)**	**37637 (98.26)**	**666 (1.74)**	**38303 (100)**		
**R-J classification of Index case**	**I/TT/BT/PN**	35398 (98.77)	441 (1.23)	35839 (100)	842.37	<0.001
	**BB/BL/LL**	2239 (90.87)	225 (9.13)	2464 (100)		
	**Total (%)**	**37637 (98.26)**	**666 (1.74)**	**38303 (100)**		
**WHO grade of Index case**	**0**	33371 (98.44)	529 (1.56)	33900 (100)	55.02	<0.001
	**1**	2213 (96.89)	71 (3.11)	2284 (100)		
	**2**	2049 (96.88)	66 (3.12)	2115 (100)		
	**Total (%)**	**37633 (98.26)**	**666 (1.74)**	**38299 (100)**		
**Initial smear of Index case**	**0**	32177 (98.76)	405 (1.24)	32582 (100)	927.97	<0.001
	**1–3 (low positive)**	637 (95.5)	30 (4.5)	667 (100)		
	**4–6 (high positive)**	1307 (88.31)	173 (11.69)	1480 (100)		
	**Total (%)**	**34121 (98.25)**	**608 (1.75)**	**34729 (100)**		

**Table 3:** Results of FU for HH contacts for all related variables, including Chi^2^ test results.

Variable		Number of HH contacts		Total (%)	Chi^2^	P value
		No leprosy (%)	Leprosy (%)			
**Blood Relative**	**Unrelated**	8521 (98.08)	167 (1.92)	8688 (100)	2.21	0.137
	**Related**	29116 (98.32)	499 (1.68)	29615 (100)		
	**Total**	**37637 (98.26)**	**666 (1.74)**	**38303 (100)**		
**Index duration of symptoms (months)**	**Up to 12**	26707 (98.25)	475 (1.75)	27182 (100)	0.233	0.89
	**13–24**	5227 (98.23)	94 (1.77)	5321 (100)		
	**25+**	5679 (98.34)	96 (1.66)	5775 (100)		
	**Total**	**37613 (98.26)**	**665 (1.74)**	**38278 (100)**		
**Duration of HH contact (years)**	**0–5**	8012 (99.22)	63 (0.78)	8075 (100)	55.03	<0.001
	**6+**	29625 (98.01)	603 (1.99)	30228 (100)		
	**Total**	**37637 (98.26)**	**666 (1.74)**	**38303 (100)**		
**Previous diagnosed count**^a^	**0**	36449 (98.34)	615 (1.66)	37064 (100)	44.82	<0.001
	**1**	1057 (96.09)	43 (3.91)	1100 (100)		
	**2+**	131 (94.24)	8 (5.76)	139 (100)		
	**Total (%)**	**37637 (98.26)**	**666 (1.74)**	**38303 (100)**		
**Co-prevalent diagnoses**^b^	**0**	37083 (98.86)	429 (1.14)	37512 (100)	42000	<0.001
	**1**	512 (73.56)	184 (26.44)	696 (100)		
	**2+**	42 (44.21)	53 (55.79)	95 (100)		
	**Total (%)**	**37637 (98.26)**	**666 (1.74)**	**38303 (100)**		

^a^ number of HH contacts diagnosed before the index case diagnosis.

^b^ count of HH contacts diagnosed at year zero, effectively concurrent with the index case.

There was a significant association (p<0.001) between development of leprosy amongst HH contacts and smear positivity, leprosy group/classification and sex of index case and WHO disability grade of the index case. Significant risk factors (p<0.001) relating to individual HH contacts and the development of leprosy amongst HH contacts were presence of contacts previously diagnosed or co-prevalent cases, HH contacts diagnosed immediately after the index diagnosis (year 0), duration of HH contact and age of HH contact at index case diagnosis.

Out of the HH contacts of index cases with high positive smear (1480), 11.69% were diagnosed with leprosy throughout the follow up. This is compared to HH contacts of smear negative index cases (32582), where 1.24% were diagnosed with leprosy, supporting our findings of a significant association between level of smear of index case and risk of leprosy development.

Looking at MB/PB classification of the index case– 4.4% of HH contacts of index cases who were diagnosed with MB leprosy developed leprosy throughout the follow-up compared to 1.08% of HH contacts of PB index cases.

Table 4 shows that index smear, WHO grade, Ridley-Jopling classification and leprosy group were independently associated with increased risk of leprosy development amongst HH contacts after stepwise analysis using cox proportional hazard regression.

**Table 4:** Results of cox proportional hazard regression analysis.

Variables relating to index cases	Univariate analysis		Multivariate analysis		After stepwise pr analysis	
	Hazard ratio (p value)	95% CI^a^	Hazard ratio (p value)	95% CI	Hazard ratio (p value)	95% CI
Index smear	3.47 (<0.001)	3.12, 3.85	1.57 (<0.001)	1.29, 1.91	1.57^c^ (<0.001)	1.29, 1.91
Index WHO grade	1.62 (<0.001)	1.42, 1.86	1.18 (0.036)	1.01, 1.38	1.17 (0.043)	1.01, 1.37
Index child count	1.42 (<0.001)	1.18, 1.71	0.57 (<0.001)	0.45, 0.74	0.6 (<0.001)	0.47, 0.76
Index leprosy group	5.2 (<0.001)	4.33,6.24	2.1 (<0.001)	1.58, 2.81	2.1 (<0.001)	1.57, 2.8
Index RJ classification	9.88 (<0.001)	8.18, 11.94	2.82 (<0.001)	1.91, 4.2	2.81 (<0.001)	1.9, 4.14
Index sex	0.73 (0.001)	0.6, 0.88	1.04 (0.659)	0.84, 1.3	NS^b^	NS
Index duration of symptoms (diagnostic delay)	0.93 (0.26)	0.82, 1.05	0.92 (0.215)	0.8, 1.05	NS	NS
**Variables relating to HH contacts**						
Contact sex	1.03 (0.734)	0.86, 1.24	0.97 (0.773)	0.79, 1.19	NS	NS
Contact age group	1.42 (<0.001)	1.18, 1.7	1.1 (0.414)	0.99, 1.2	NS	NS
Blood relative	0.796 (0.03)	0.65, 0.98	0.84 (0.169)	0.65, 1.08	NS	NS
**Variables relating to HH**						
Duration of HH contact	2.29 (<0.001)	1.7, 3.07	2.04 (<0.001)	1.49, 2.8	2.01 (<0.001)	1.46, 2.75
Previous diagnosis count	1.89 (<0.001)	1.42, 2.52	1.41 (0.042)	1.01, 1.95	1.4 (0.045)	1.01, 1.94
Co-prevalent count	3.81 (<0.001)	2.9, 5.01	1.97 (<0.001)	1.45, 2.69	1.95 (<0.001)	1.43, 2.66

^a^ CI = confidence interval

^b^ NS = not significant (p<0.05)

^c^ Index smear was analysed in three groupings–smear negative, low positive (1–3) and high positive. (4–6) (Tables 1 and 2). A hazard ratio of 1.57 for Index smear relates to a 1.57 increased risk of leprosy development per group increase of smear level. HH contacts of highly smear positive index cases are at 3.14 times increased risk of leprosy development compared to HH contacts of smear negative index cases

As the initial smear of the index case increases from negative to low positive (1–3) to high positive (4–6), the hazard of their associated HH contacts developing leprosy increases by 1.57 times (p<0.001). Contacts of high smear positive index cases are at 3.14 times increased risk compared to contacts of smear negative index cases. For every one unit increase in WHO grade of the index case, the hazard of HH contacts developing leprosy increases by 1.17 times. (p = 0.043) A hazard ratio of 2.1 (p<0.001) for index leprosy group suggests that HH contacts of an index case who has MB compared to PB leprosy results in 2.1 times increased risk of developing leprosy. HH contacts of index cases with BB/BL/LL (lepromatous leprosy) index cases are at 2.81 times (p<0.001) increased risk of developing leprosy compared to HH contacts of index cases with I/TT/BT/PN (non-lepromatous) leprosy. Looking only at the variables available for analysis, the level of initial smear of the index case is associated with the highest level of risk for their associated HH contact developing leprosy.

A hazard ratio of 0.6 (p<0.001) for index child count means HH contacts of adult index cases (aged 16+ years) are at 40% reduced hazard of leprosy development compared with HH contacts of child index cases (aged 0–15 years). Duration of HH exposure, previous diagnosis count and co-prevalent count were all independently associated with an increased risk of leprosy development.

For every one person increase in the number of co-prevalent diagnoses (0/1/2+), the hazard of HH contacts developing leprosy increases by 1.95 times (p<0.001). As the duration of HH contact goes from 0–5 to 6+ (years), the hazard of HH contact developing leprosy increases by 2.01 times (p<0.001). As the previous diagnoses count (number of HH contacts diagnosed before the index diagnosis) within one HH increases by a factor of one, (0/1/2+) the hazard of HH contacts developing leprosy increases by 1.4 times (p = 0.045).

## Discussion

Of the variables available for analysis, this study shows a surprising dominance of a single variable predicting risk of the development of leprosy amongst HH contacts–skin smear positive index cases. This has significant public health and patient implications. It is interesting that a single variable is so dominant in determining risk of leprosy development amongst HH contacts in Bangladesh which has a very high PB rate, the proportion of leprosy types in this study is typical for NW- Bangladesh. This study should be repeated in a setting with a higher proportion of MB cases.

We are confident of these findings because this is the largest, most comprehensive study of development of leprosy in HH contacts conducted in recent years and has a long mean follow-up period. The identification of a single, dominant risk factor simplifies contact tracing and allows the prioritization of HH contact examinations.

A small number of index cases seem to be maintaining leprosy transmission in Bangladesh. Targeting HHs of skin smear positive index cases for contact examinations and detecting new smear positive cases is a more efficient allocation of restricted resources.

Of the variables analysed in this study, smear positivity stands out as a single, dominant variable predicting risk of leprosy development amongst HH contacts. HH contacts of highly smear positive index cases were found to be at 3.14 times increased risk of leprosy development compared to HH contacts of index cases who are smear negative. (Table 4) A hazard ratio of 2.81 and 2.10 for HH contacts of BB/BL/LL and MB index cases, respectively, was found. A difference in risk to HH contact was found depending upon level of initial smear of index case (smear negative, low positive, high positive). (Table 4) Conducting skin smear tests upon diagnosis is important for classifying patients and for appropriate treatment allocation with either PB or MB chemotherapy and our results emphasize the value of training health staff to accurately classify leprosy. However, these results also show its importance as a predictor for risk of leprosy development amongst HH contacts. 11.69% of HH contacts of highly smear positive index cases were diagnosed with leprosy throughout the follow up, compared to 1.24% of HH contacts of smear negative index cases. (Table 2) Slit-skin smear services are not always available and quality control of smear reading is sometimes lacking. Slit-skin smears should be conducted on every new case detected in Bangladesh, and everywhere, for risk identification and appropriate allocation to MB/PB or RJ classification. Training for field staff needs to be given and adequate resources need to be provided.

The COCOA study followed a large number of subjects in a high leprosy prevalence region. Because of good quality routine record keeping in a leprosy control unit within a high population density area, retrospective data was of good quality and a high level of completeness. The diagnostic accuracy is expected to be good as this study was conducted in a control unit that has been engaged in clinical and epidemiological leprosy research for the last twenty years.

However, during the study period, fieldworkers may not have assessed all HH contacts during the HH visits. There is limited information on HHs that were not assessed at all during the study period due to time constraints; 62.59% of all eligible HHs who met inclusion criteria were enrolled. There is no reason to believe that the HHs not examined differed systematically in terms of index case profile and HH features compared to HHs who were examined. No data was collected on history of receiving BCG vaccination nor socio-economic status of the HH. [27] Higher SES is associated with protection against leprosy. [10, 28] These factors should be examined in future research. This was a secondary data analysis of the COCOA study, which sought to provide evidence for timings of contact examination policies. The risk factors examined in this analysis were therefore restricted to the variables collected in the original study. No risk factors directly relating to community and environmental transmission, nor socio-economic data pertaining to the HH, were collected in the COCOA study, so the role of environment in leprosy transmission could not be analysed. Leprosy transmission seems to be more complicated than only human-to-human transmission; the significance of environmental reservoirs in leprosy transmission is currently not well-understood. [22] It is imperative that future prospective cohort studies looking at risk factors for leprosy development include variables and potential transmission reservoirs relating to both the HH and the surrounding environment.

Studies in Brazil [29], Philippines [30] and Malawi [31] show that HH contacts of MB index cases are at increased risk of leprosy development. Another Bangladeshi study showed that blood relationship, age of contact, close contact to index case and classification of index case are all independently associated with increased risk of leprosy. [16] In this analysis however, only the index case classification was significant. Similar to published studies leprosy group/classification of index case were independently associated with increased risk of leprosy (Table 4), consistent with the consensus that MB and lepromatous leprosy patients are important sources of leprosy transmission. [31]

Looking at whether the type of index case influences the classification of the contacts, amongst co-prevalent cases a higher proportion of MB amongst new cases was observed when the index case is MB. [22]

HH contacts residing in HHs with two or more co-prevalent diagnoses were at 3.9 times increased risk of leprosy development compared to HH contacts residing in HHs where there were no concurrent cases diagnosed at year 0. (Table 4) Current guidelines do not mention specifically targeting multiple case HHs as part of case detection, [21] however this should be made a priority and integrated into the current guidelines for performing contact examinations in Bangladesh.

Males predominated amongst index cases, 70.66% of MB index cases were male, (Table 1) this male excess has been found many times. [31] However, no significant association was found between sex of HH contact and development of leprosy. Sex and risk of leprosy development are not well understood. [18] Several factors may contribute to the higher adult male rate including men being at higher risk through being outside the home and case ascertainment with female cases not being found by male health workers. Cultural factors may be important.

We expected that blood relatives would be at an increased risk of leprosy development with a shared environment and potential genetic susceptibility [32], but none was found. Participants were asked a binary response to the genetic relationship. A more detailed definition of blood relative might have allowed detection of a significant association.

Interestingly we found a difference in risk for leprosy development amongst HH contacts depending on whether the index case is a child or adult. HH contacts of adult index cases are at a 40% reduced risk of leprosy development compared to contacts of child index cases. (Table 4) Child index cases were found to be more PB as described by Butlin et al. [22] Closeness of contact and social factors may be significant. Child index cases inherently require more care and close contact from HH members, which could be a factor in this increased risk.

Surprisingly index duration of symptoms before diagnosis (i.e. diagnostic delay) did not increase the risk of HH contacts developing leprosy despite close and prolonged contact of an untreated index case being a major determinant of transmission. [15] The quality and accuracy of this variable is dependent on the ability of the index case to accurately recall time of first symptom, potentially introducing bias due to the sometimes inconspicuous initial clinical presentation. Perhaps duration of HH exposure is more useful in terms of predicting risk of leprosy development than index duration of symptoms, as the information may be more reliable. Duration of HH contact was divided into two categories; up to 5 years and more than 5 years. There was no significant change in RJ classification when the duration of HH contact was more than 5 years compared to up to 5 years. (p = 0.926).

The administration of SDR to household and close contacts of leprosy patients is a prophylactic intervention used for leprosy control. Our study found that HHs of smear positive index cases are at a far increased risk compared to HHs of smear negative index cases. (Hazard ratio– 3.14, p<0.001) (Table 4). 11.69% of HH contacts of high smear positive index cases developed leprosy, compared to only 1.24% of HH contacts of smear negative index cases that were diagnosed with leprosy. (Table 2) If a small number of MB smear positive patients are predominantly sustaining leprosy infection within the HH setting, the prophylactic intervention of administering SDR to contacts will not solely succeed because SDR was only found to interrupt transmission in households of PB index cases. [33]

Our results highlight that SDR administration for HH contacts of confirmed index cases will not be solely effective. We suggest a different emphasis for routine HH contact examinations. Performing slit skin smears is necessary for every newly diagnosed case for appropriate allocation to PB/MB regimen, and the results of this study have highlighted its importance in predicting risk. Moreover, as we have identified a single, dominant risk factor for the development of leprosy, we are suggesting routine HH contact examinations should be focused on HHs of smear positive index cases to increase the efficiency of this control effort and case finding; with limited economic and human resources this seems the most effective direction for future efforts.

This is one of the largest cohort studies to ever be conducted with a very long follow up period of 21 years, and we have found a single, dominant risk factor for leprosy development amongst HH contacts–smear positivity. This study shows that contact examinations in Bangladesh should target HHs of skin smear positive index cases to detect most new cases and interrupt transmission. Slit-skin smear services need be given adequate resources to identify the infectious smear positive patients as a predictor for risk of leprosy development amongst HH contacts. Furthermore, the use of SDR in Bangladesh as a prophylactic intervention needs to be re-examined in light of our results.
